# Negative correlation between relative fat mass and bone mineral density: NHANES 2011–2018

**DOI:** 10.3389/fpubh.2025.1584293

**Published:** 2025-07-03

**Authors:** Xing-An Jiang, Hong-Li Zhang, Bin-Tao Ye, Shao-Qi Fang, Jia-Jin Zhang, Hao-Lin Yu, Jun-Bo Liang

**Affiliations:** ^1^Department of Orthopedic, Taizhou Hospital Affiliated to Zhejiang University School of Medicine, Taizhou, China; ^2^School of Medicine, Zhejiang University, Hangzhou, China; ^3^Orthopedic Department, Taizhou Hospital Affiliated to Wenzhou Medical University, Taizhou, China; ^4^School of Medicine, Shaoxing University, Shaoxing, China

**Keywords:** relative fat mass, bone mineral density, osteoporosis, NHANES, obesity

## Abstract

**Background:**

Utilizing data from the National Health and Nutrition Examination Survey (NHANES), this study investigated the association between Relative Fat Mass (RFM) and lumbar spine bone mineral density (BMD) in adults, specifically evaluating the impact of RFM on lumbar BMD and determining the consistency of this relationship across diverse populations.

**Methods:**

Cross-sectional data from 9,238 adults aged ≥20 years in the 2011–2018 NHANES cycles were analyzed. Relative fat mass (RFM) values were derived through a formula incorporating waist circumference (WC) and height. The relationship between RFM and lumbar BMD was assessed through weighted multiple linear regression models, subgroup analyses, and smooth curve fitting.

**Results:**

After adjusting for multiple covariates, RFM exhibited a statistically significant inverse relationship with lumbar spine BMD. In the fully adjusted model, a per-unit increment in RFM was linked to a decline of 0.0110 g/cm^2^ in lumbar BMD (β = −0.0110, 95% CI: −0.0132 to-0.0088; *p* < 0.0001). Additionally, an inflection point was detected at RFM = 20.5847 (*p* < 0.001), with significantly distinct correlations between RFM and lumbar spine BMD when values were above or below this point. Subgroup analyses confirmed the persistence of this inverse relationship in virtually all population subgroups stratified by demographic characteristics or health statuses.

**Conclusion:**

The analysis reveals a notable inverse relationship between RFM values and BMD measurements in the lumbar spine, suggesting that elevated RFM levels might correlate with reduced BMD and heightened susceptibility to osteoporosis (OP) development. These observations highlight the critical role of evaluating adipose distribution patterns when devising preventive measures against OP and support employing RFM as an potential indicator for initiating early clinical interventions aimed at mitigating bone density deterioration.

## 1 Background

Osteoporosis (OP) constitutes a significant global health challenge characterized by low BMD and compromised bone tissue microstructure, resulting in heightened susceptibility to skeletal fractures (1). According to WHO criteria, osteoporosis can be diagnosed based on BMD measurements with a T-score ≤ − 2.5 SD (2). With ongoing demographic shifts toward older age groups, the socioeconomic impact of osteoporosis-related complications continues to grow substantially across healthcare systems worldwide (3).

Excessive adiposity has emerged as a major public health challenge, classified as a persistent endocrine dysfunction characterized by abnormal lipid deposition and systemic metabolic dysregulation (4). Since the late 20th century, worldwide rates of excessive weight have more than doubled, currently affecting approximately 40% of adults globally (5). The condition imposes substantial economic burdens, contributing to annual healthcare costs exceeding $150 billion in some nations (6), while concurrently elevating risks for metabolic derangements, cardiovascular pathologies, and neoplastic developments (5). BMI remains the predominant anthropometric index in clinical practice owing to its operational simplicity. Nevertheless, its shortcomings are now well-documented; this metric fails to differentiate lean body mass from adipose tissue and remains vulnerable to measurement inaccuracies across diverse demographic groups (7).

Proposed by Orison et al., RFM represents an innovative approach to evaluating obesity through a straightforward mathematical formula derived from waist circumference and height measurements. Designed for individuals aged 20 years or older, this metric offers a viable substitute method for assessing overall adiposity levels in both male and female populations. Empirical studies confirm its applicability across diverse ethnic groups, with evidence indicating superior accuracy in evaluating total body fat compared to traditional BMI (8). Additionally, RFM demonstrates significant correlations with multiple disorders, such as type 2 diabetes mellitus (T2DM), hypertension, and coronary artery disease (CAD), as highlighted in prior research (9–11). These findings underscore its clinical utility, suggesting that RFM may deliver enhanced precision in quantifying adiposity while improving risk prediction and mitigation strategies for obesity-associated comorbidities in healthcare settings.

Current research has not yet confirmed correlations between RFM and lumbar spine BMD. This cross-sectional analysis leverages datasets from NHANES to examine potential connections between these variables within the American population aged ≥20 years.

## 2 Methods

### 2.1 Study population

As a nationally recognized health assessment program, the National Health and Nutrition Examination Survey (NHANES) is administered by the National Center for Health Statistics (NCHS) as a population-based cross-sectional research initiative. To ensure methodological rigor and demographic diversity, the survey utilizes a multiphase probability sampling framework with stratification and multistage selection processes. Ethical oversight for NHANES protocols was granted by the NCHS review committee, with all participants having obtained documented informed consent for the anonymized use of their health data in scientific analyses (12).

**Figure 1:**
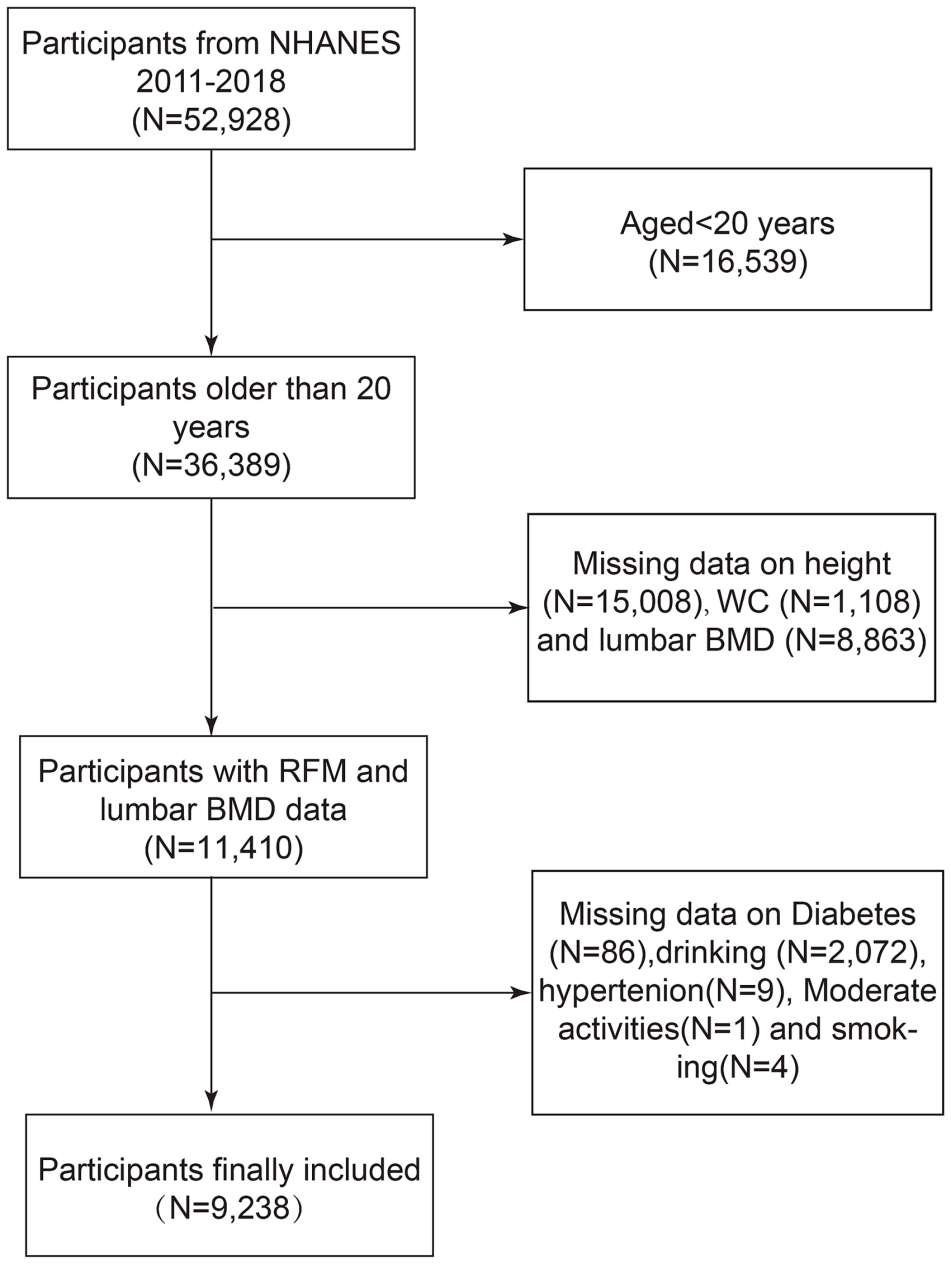
Flow chart of participants selection. NHANES, National Health and Nutrition Examination Survey; WC, Waist Circumference; BMD, Bone Mineral Density; RFM, Relative fat mass.

The analysis draws on datasets collected during NHANES cycles spanning 2011 to 2018, with an original cohort of 52,928 American subjects. The dataset included health metrics, dietary records, laboratory test results, and questionnaire responses. Specific exclusion parameters were implemented sequentially: participants under 20 years of age (*N* = 16,539), those missing height (*N* = 15,008) or WC data (*N* = 1,108), individuals without lumbar spine BMD data (*N* = 8,863), and those with missing data on diabetes history (*N* = 86), alcohol consumption history (*N* = 2,072), hypertension history (*N* = 9), moderate physical activity questionnaire data (*N* = 1), or smoking history (*N* = 4). Following these exclusions, 9,238 qualified individuals were retained for subsequent analytical evaluation (Figure 1).

### 2.2 Study variables

BMD in the lumbar region was quantified through dual-energy X-ray absorptiometry (DXA), conducted by qualified radiologic technicians employing a Hologic QDR 4500 A device paired with Apex version 3.2 software (13). Relative Fat Mass (RFM), an anthropometric index for body fat estimation, was derived from the formula RFM = 64 − (20 × height/waist circumference) + (12 × sex), where sex = 0 for males and sex = 1 for females (8). Participants completed specific health questionnaires covering medical history, medication use, smoking status, alcohol consumption, and physical activity levels. Height and WC were measured by trained health professionals at the Mobile Examination Center (MEC). Height was measured using a specialized stadiometer, with participants standing barefoot, heels together, back against the board, and head positioned horizontally. WC was determined by marking a horizontal reference line superior to the right ilium’s uppermost lateral edge, followed by positioning a measurement tape at the intersection of these anatomical markers.

In this study, we evaluated various covariates, including age, sex, race(including Mexican American, other Hispanic, Non-Hispanic white, Non-Hispanic black, or other.), education level(ranging from Less than 9th grade to College graduate or above, covering 9-11th grade, High School, and Some college or AA degree), poverty income ratio (PIR), weight, body mass index (BMI), triglycerides (TG), total cholesterol (TC), high-density lipoprotein cholesterol (HDL-C), vitamin D3 (25-OHD 3), total calcium, serum phosphorus, blood urea nitrogen, serum uric acid, creatinine, alanine aminotransferase (ALT), aspartate aminotransferase (AST) levels, hypertension status(ever told you had high blood pressure), diabetes status(self-reported diabetes, current use of insulin or other glucose-lowering medications, or an HbA1c level of 6.5% or higher, or fasting plasma glucose exceeding 7 mmol/L), smoking status(having smoked at least 100 cigarettes in a lifetime), alcohol consumption history(having consumed at least 4 drinks per day for women or 5 drinks per day for men), and moderate physical activity levels(having moderate-intensity sports, fitness, or recreational activities for at least 10 min continuously in a typical week).

### 2.3 Data analysis

In consideration of NHANES’s intricate multistage probability sampling methodology, appropriate weighting and variance estimation methods were utilized to ensure accurate statistical inferences. Following stratification of RFM into tertiles, the association between RFM and lumbar spine BMD was examined through a multivariate logistic regression approach, incorporating sampling weights. For continuous variables, weighted linear regression analyses were conducted to assess intergroup disparities, while categorical variables were evaluated using weighted chi-square tests. To determine the independent relationship between RFM and lumbar spine BMD, three models were developed. The first model employed a weighted univariate linear regression framework. Subsequent models (2 and 3) adopted weighted multivariate linear regression methodologies. Model 2 accounted for demographic covariates, including sex, age, and racial/ethnic background. Model 3 extended these adjustments to education level, PIR, weight, BMI, TG, TC, HDL-C, 25-OHD 3, total calcium, serum phosphorus, blood urea nitrogen, serum uric acid, creatinine, ALT, AST levels, hypertension status, diabetes status, smoking status, alcohol consumption history, and moderate physical activity levels.

Stratified multivariate regression analysis was conducted for subgroup analysis. Participants were stratified according to multiple variables encompassing age, sex, hypertension history, diabetes history, smoking history, alcohol consumption history, moderate physical activity, BMI, and race/ethnicity. The nonlinear relationship between RFM and lumbar spine BMD was explored using smooth curve fitting and generalized additive models. Specifically, a recursive algorithm was used to identify inflection points in the relationship between RFM and lumbar spine BMD, and a two-piece linear regression model was applied on either side of the inflection point. Subgroup analysis and log-likelihood ratio tests were used to evaluate interactions between subgroups. For missing data, continuous variables were imputed using median or mean values based on data distribution. Analyses were performed using R software and Empower software, with statistical significance defined as a *p*-value less than 0.05.

## 3 Results

### 3.1 Participant characteristics

The study included 9,238 participants with a mean age of 39.22 years, of whom 45.67% were female. Based on RFM levels, participants were divided into tertiles: low (7.756–29.604), medium (29.604–38.179), and high (38.179–56.675) (Table 1). Relative to the lowest RFM tertile group, individuals in the upper tertile showed increased probabilities to be female, older, and have a college or AA degree. Additionally, the highest RFM demonstrated elevated hypertension prevalence and reduced participation in moderate activities, but lower rates of smoking, alcohol consumption, and diabetes. In analyses of anthropometric and metabolic indicators, the highest RFM tertile displayed elevated values in BMI, weight, waist circumference, total cholesterol, and HDL-C levels, while height, total calcium, creatinine, blood urea nitrogen, serum uric acid, and AST levels were lower.

**Table 1:** Basic characteristics of the study population based on RFM tertiles.

RFM (%)	Low (*n* = 3,079)	Medium (*n* = 3,079)	High (*n* = 3,080)	*p*-value
	(7.756，29.604)	(29.604，38.179)	(38.179,56.675)	
Age (years)	36.9989 ± 11.8015	39.8287 ± 11.6349	41.5611 ± 11.2732	<0.0001
Sex, *n* (%)				<0.0001
Male	96.8497	59.8421	4.2299	
Female	3.1503	40.1579	95.7701	
Race/ethnicity (%)				<0.0001
Mexican American	8.0467	10.6246	10.5336	
Other Hispanic	6.5052	7.4262	6.6998	
Non-Hispanic White	63.4627	65.2374	61.7647	
Non-Hispanic Black	11.6124	8.4751	13.9394	
Other Race	10.3730	8.2367	7.0624	
Education level (%)				<0.0001
Less than 9th grade	2.9117	3.2749	2.7586	
9-11th grade	2.9117	3.2749	2.7586	
High school	22.5063	21.3117	21.4825	
Some college or AA degree	29.0916	32.2883	38.3302	
College graduate or above	35.4757	34.5434	28.2294	
Income to poverty ratio (PIR)	3.0148 ± 1.6352	3.1016 ± 1.6202	2.8222 ± 1.6126	<0.0001
Moderate activities (%)				<0.0001
Yes	52.7710	48.8537	46.5782	
No	47.2290	51.1463	53.4218	
Smoking (%)				0.0243
Yes	46.2607	45.3421	42.8760	
No	53.7393	54.6579	57.1240	
Drinking (%)				<0.0001
Yes	18.5506	17.9921	10.5187	
No	81.4494	82.0079	89.4813	
Diabetes, *n* (%)				<0.0001
Yes	3.6254	9.0518	12.1036	
No	96.3746	90.9482	87.8964	
Hypertension, *n* (%)				<0.0001
Yes	17.1419	23.5960	28.2344	
No	82.8581	76.4040	71.7656	
BMI (kg/cm^2^)	24.9893 ± 3.2735	28.8802 ± 6.2475	33.2362 ± 7.4834	<0.0001
Height (cm)	176.2753 ± 7.3645	171.0219 ± 8.9267	162.8950 ± 7.1139	<0.0001
Weight (kg)	77.9330 ± 12.9142	85.8332 ± 24.7155	88.6847 ± 23.2860	<0.0001
WC (cm)	89.6785 ± 9.4413	99.4866 ± 17.9733	106.4162 ± 16.8169	<0.0001
TG(mg/dl)	143.6190 ± 150.1643	162.9787 ± 149.8353	147.8058 ± 105.1804	<0.0001
Total cholesterol (mg/dl)	187.7089 ± 40.1629	192.3025 ± 38.7978	196.9051 ± 39.9689	<0.0001
HDL-C (mmol/L)	1.3409 ± 0.3733	1.3608 ± 0.4560	1.3906 ± 0.3696	<0.0001
25OHD3 (nmol/L)	64.3134 ± 23.5766	65.8632 ± 27.0184	61.6267 ± 27.4203	<0.0001
Total calcium (mg/dl)	9.4516 ± 0.3166	9.3593 ± 0.3181	9.3089 ± 0.3463	<0.0001
Phosphorus (mg/dl)	3.6935 ± 0.5523	3.7181 ± 0.5531	3.7114 ± 0.5297	0.1843
Creatinine (mg/dl)	0.9570 ± 0.1864	0.8728 ± 0.2572	0.7511 ± 0.1912	<0.0001
Blood urea nitrogen (mg/dl)	13.5704 ± 4.0102	12.9602 ± 4.1346	12.0205 ± 4.1905	<0.0001
Serum uric acid (μmol/L)	342.1862 ± 68.8097	324.7792 ± 90.6286	296.6150 ± 71.5527	<0.0001
ALT (U/L)	27.2312 ± 20.4411	28.2669 ± 20.5079	23.0558 ± 16.6520	<0.0001
AST (U/L)	26.5334 ± 16.0458	25.5118 ± 16.6235	23.5484 ± 16.6680	<0.0001
Lumbar spine BMD (kg/cm^2^)	1.0507 ± 0.1516	1.0320 ± 0.1524	1.0399 ± 0.1428	<0.0001

Presented using mean values and their 95% confidence intervals for continuous variables, and percentage frequencies and their 95% confidence intervals for categorical variables. PIR, poverty income ratio; BMI, body mass index; WC, waist circumference; TG, triglycerides; HDL-C, high-density lipoprotein cholesterol;25-OHD3, vitamin D3; ALT, alanine aminotransferase; AST, aspartate aminotransferase; BMD, bone mineral density.

### 3.2 Weighted multivariate linear regression analysis

The analytical framework incorporated covariate-adjusted multivariate regression models to evaluate associations between RFM and lumbar spine BMD (Table 2). Initial unadjusted analyses demonstrated an inverse association (β = −0.0007, 95% CI: −0.0010, −0.0003), which persisted robustly in subsequent models. Following incremental covariate adjustment, Model 2 (β = −0.0018, 95% CI: −0.0024, −0.0013) and Model 3 (β = −0.0110, 95% CI: −0.0123, −0.0096) maintained statistical significance. Stratified analyses of RFM tertiles revealed that in the fully adjusted Model 3, the uppermost tertile exhibited a marked reduction in lumbar spine BMD (β = −0.0573 g/cm^2^, 95% CI: −0.0733, −0.0413) relative to the lowest tertile, with stringent statistical significance (*p* < 0.0001).

**Table 2:** Association between RFM and Lumbar spine BMD.

Exposure	Model 1 β (95% CI), *p*-value	Model 2 β (95% CI), *p*-value	Model 3 β (95% CI), *p*-value
RFM (continuous)	−0.0007 (−0.0010, −0.0003) 0.000136	−0.0018 (−0.0024, −0.0013) <0.000001	−0.0110 (−0.0123, −0.0096) < 0.000001
RFM(quartile)			
Low	Reference	Reference	Reference
Medium	−0.0186 (−0.0260, −0.0112) < 0.000001	−0.0224 (−0.0305, −0.0143) <0.000001	−0.0437 (−0.0530, −0.0344) < 0.000001
High	−0.0108 (−0.0183, −0.0033) 0.005011	−0.0323 (−0.0439, −0.0208) <0.000001	−0.0573 (−0.0733, −0.0413) <0.000001
*p* for trend	<0.001	<0.001	<0.001

Model 1 was unadjusted; Model 2 was adjusted for sex, age, and ethnicity; and Model 3 further adjusted for education, PIR, drinking, HDL, hypertension, moderate activities, ALT, AST, blood urea nitrogen, Total calcium. Creatinine. Phosphorus, Serum uric acid, TG, smoking, Total cholesterol, 25OHD3, Diabetes, weight, BMI.

### 3.3 Smooth curve fitting demonstrating the correlation between RFM and lumbar spine BMD

**Figure 2:**
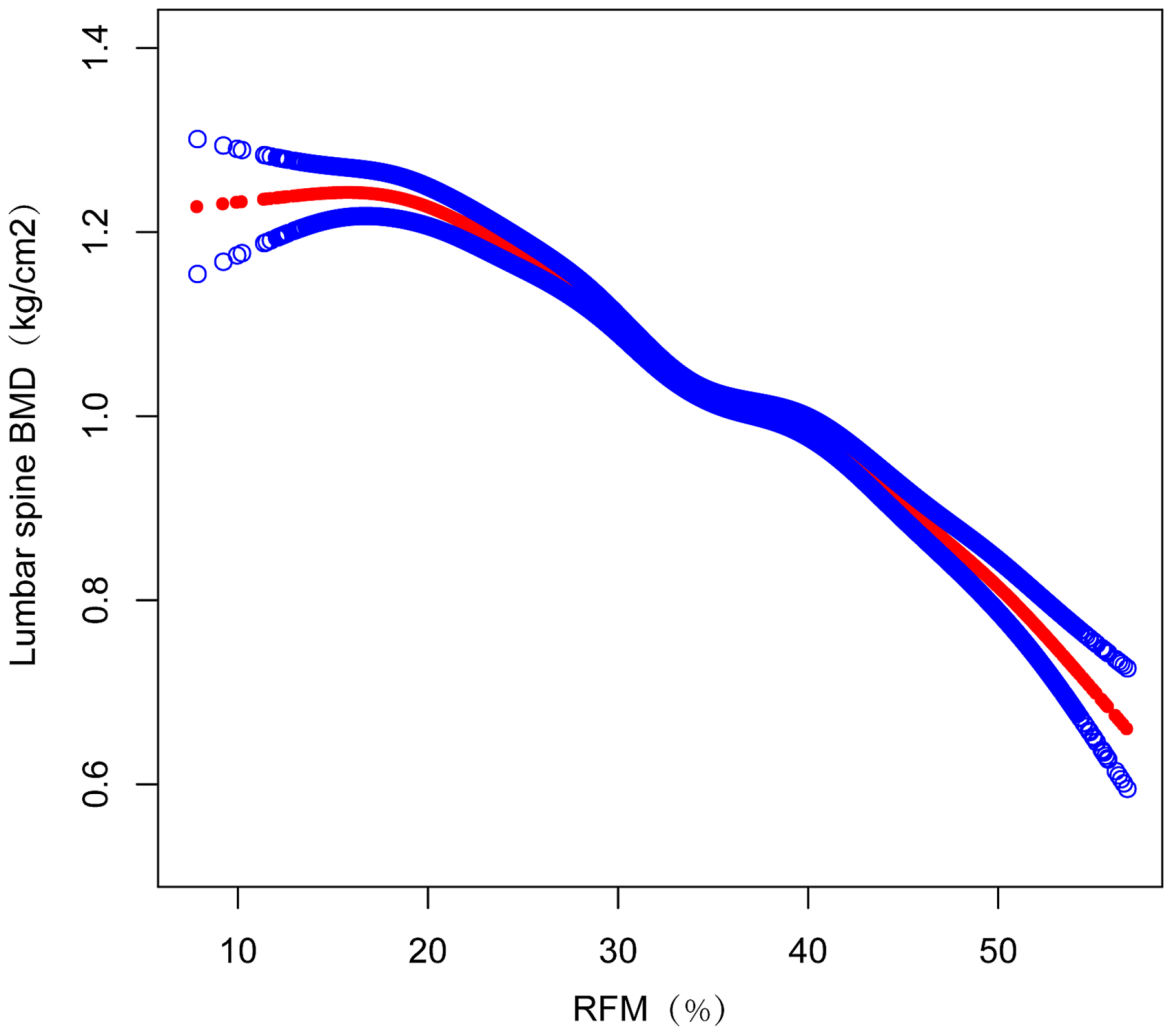
The Relationship between RFM and lumbar spine BMD. The figure shows a smooth curve fit between the variables indicated by the red line. The blue line shows the 95% confidence interval of the fit.

Nonlinear associations between RFM and lumbar spine BMD were evaluated using smooth curve fitting methodologies. These analytical approaches revealed an inverse association between RFM and lumbar spine BMD (Figure 2). Threshold effect analyses incorporating a weighted two-piece linear regression model and recursive algorithm were subsequently implemented. This approach identified a critical transition point at RFM = 20.5847 (likelihood ratio *p* < 0.001), showing stringent statistical significance. At RFM values below this threshold, a one-unit increase in RFM corresponded to an increase in lumbar spine BMD of 0.0004 g/cm^2^ (β = 0.0004 g/cm^2^, 95% CI: −0.0035, 0.0043). Above this transitional value, a one-unit increase in RFM was associated with a decrease in lumbar spine BMD of 0.0128 g/cm^2^ (β = −0.0165 g/cm^2^, 95% CI: −0.0143, −0.0113)(Table 3).

**Table 3:** Threshold effect analysis of RFM and Lumbar spine BMD.

Lumbar spine BMD	β (95% CI)	*p*-value
RFM		
Model I	−0.0110 (−0.0123, −0.0096)	<0.0001
Model II		
Inflection point (K)	20.5847	
<K point effect 1	0.0004 (−0.0035, 0.0043)	0.8409
>K point effect 2	−0.0128 (−0.0143, −0.0113)	<0.0001
Effect 2 minus effect1	−0.0132 (−0.0174, −0.0090)	<0.0001
Predicted value of the equation at the folding point	1.0489 (1.0428, 1.0551)	
Log-likelihood ratio test		<0.001

Adjusted for sex, age, race, education, PIR, drinking, HDL, hypertension, moderate activities, ALT, AST, blood urea nitrogen, Total calcium. Creatinine. Phosphorus, Serum uric acid, TG, smoking, Total cholesterol, 25OHD3, Diabetes, weight, BMI.

### 3.4 Subgroup analysis

**Figure 3:**
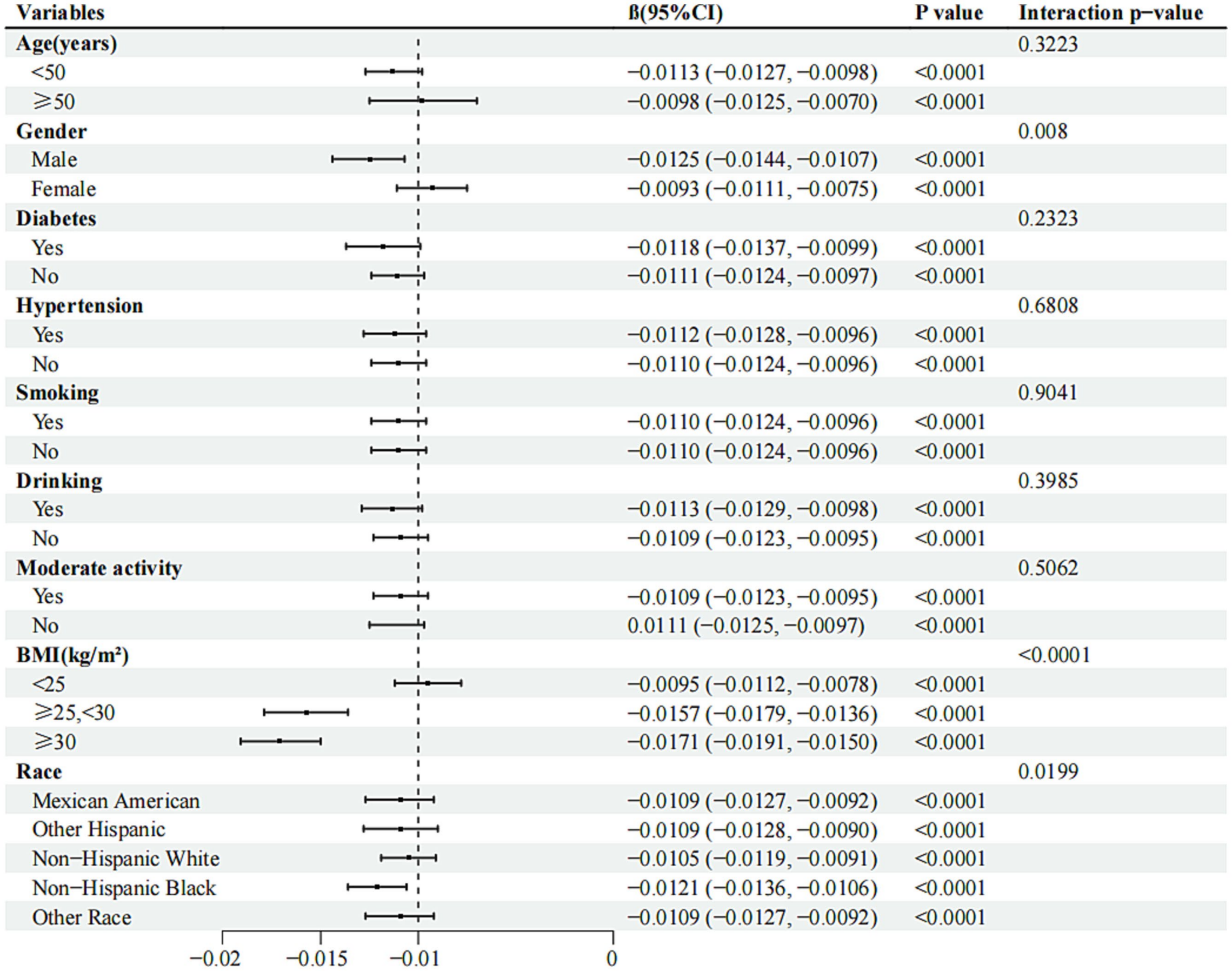
Forest plots of RFM for lumbar spine BMD in different subgroups. BMI, body mass index; CI, confdence interval.

**Figure 4:**
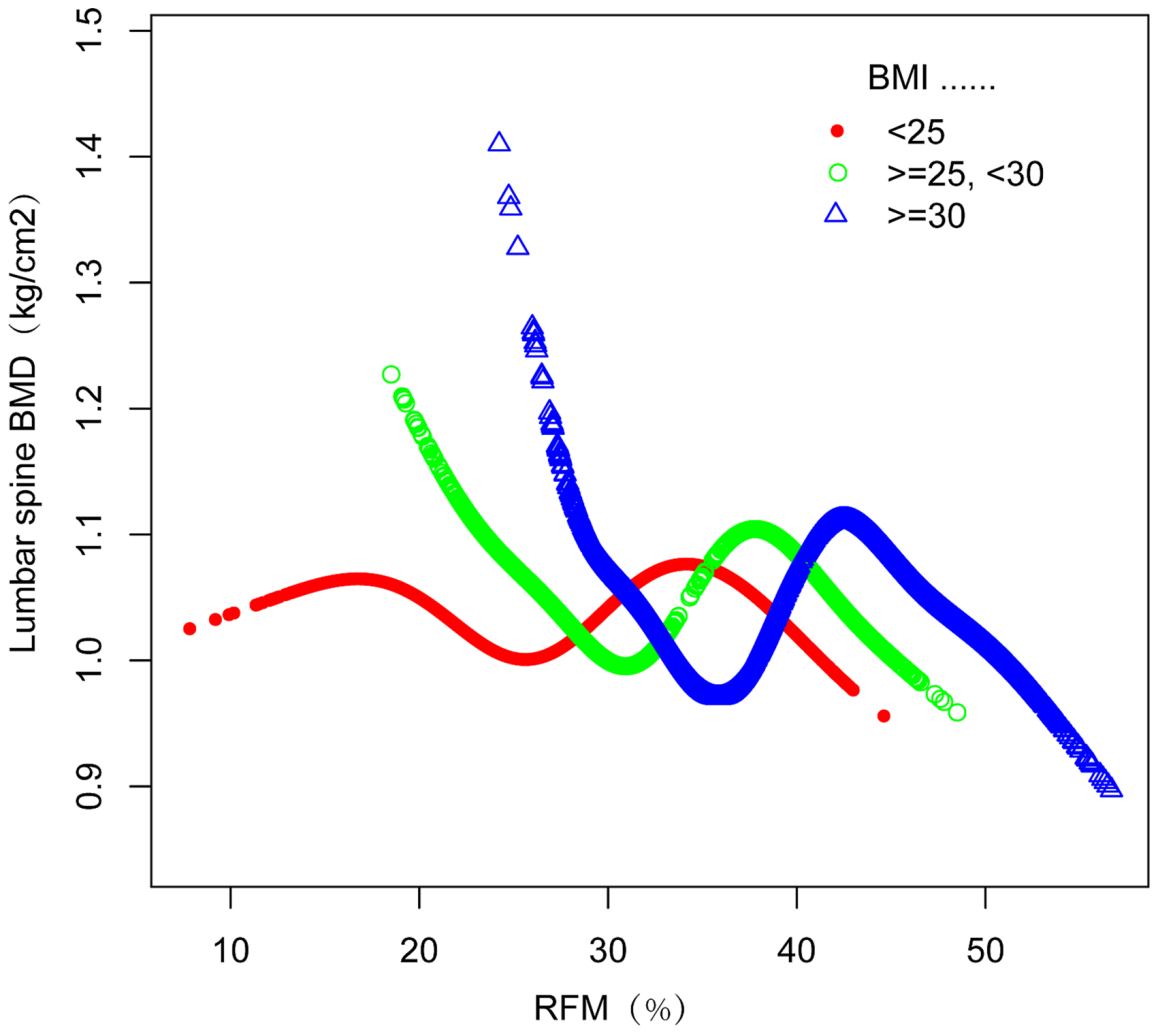
The relationship between RFM and lumbar spine BMD grouped by BMI. The subgroup analyses were performed without adjusting for BMI.

**Figure 5:**
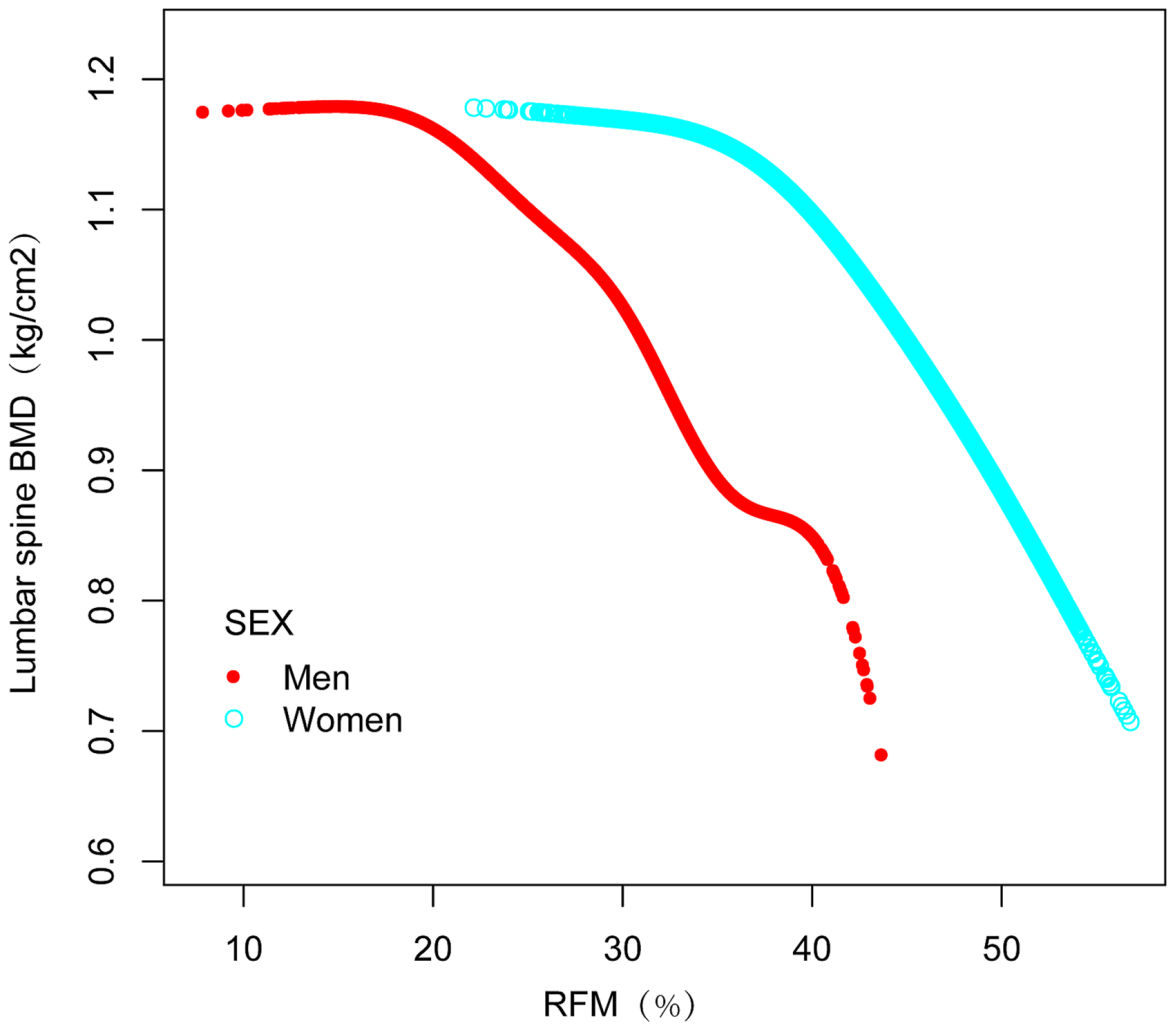
The relationship between RFM and lumbar spine BMD grouped by sex. The subgroup analyses were performed without adjusting for sex.

The investigation employed subgroup analysis and interaction tests to assess the association between RFM and lumbar spine BMD across different populations. In the fully adjusted statistical model, BMI, sex, and race/ethnicity groups showed significant interaction effects (interaction *p* < 0.05), suggesting that BMI, sex, and race/ethnicity may differentially influence the relationship between RFM and lumbar spine BMD (Figure 3). This negative correlation persisted across all relevant subgroups (*p* < 0.0001). Furthermore, smooth curve fitting was performed according to BMI and sex groups, indicating a plateau near RFM = 42 in the BMI > 30 kg/m^2^ group (Figure 4) and a plateau at RFM = 35 in male individuals (Figure 5).

## 4 Discussion

In this cross-sectional analysis involving 9,238 eligible participants, a negative association was observed between RFM and BMD at the lumbar spine, with an inflection point noted. These findings indicate managing the percentage of body fat is important for bone metabolism.

The interplay between obesity and skeletal health is multifaceted. Historically, higher body weight was viewed as a safeguard against osteoporosis, with lower BMI levels associated with an elevated risk of bone fragility and fractures (14–17). Numerous studies have demonstrated a direct association between increased BMI and higher BMD (14–19). However, the connection between body weight and bone mass does not inherently imply a causal link between obesity and osteoporosis, as obesity is characterized by excess adipose tissue rather than overall body weight. Body weight is a composite measure encompassing fat, lean muscle, and bone mass. In individuals with normal weight, fat constitutes about 16% of total body weight in men and 25% in women, with lean mass making up the majority of the remaining weight (20).

Certain studies have identified an inverse relationship between obesity and bone mineral density (BMD) (21–26). For example, Aguirre et al. observed that although BMI showed a positive association with BMD at the femoral neck, trochanter, and total body in obese individuals aged 65 or older with a BMI of 30 kg/m^2^, fat mass percentage was inversely related to BMD across all skeletal sites (21). In a cross-sectional analysis of 629 Puerto Rican adults aged 47–79, Bhupathiraju et al. noted that abdominal fat mass (AFM), adjusted for weight, was negatively associated with BMD at all four measured skeletal sites in women and at the femoral neck in men (22). Similarly, Lan et al., analyzing data from Chinese and Caucasian populations, found that after accounting for the mechanical loading effects of body weight, fat mass exhibited a negative correlation with bone mass (23). These findings underscore the importance of recognizing that body weight is composed of diverse components, and careful selection of appropriate metrics for both body weight and obesity is critical when assessing their relationship with BMD.

RFM, a straightforward and non-invasive composite measure for evaluating total body fat percentage in adults, has been shown to surpass BMI in accurately predicting body fat levels in both genders (8, 27–29). Woolcott et al. examined data from 12,581 participants in the NHANES 1999–2006 and demonstrated that RFM provided more reliable estimates of body fat percentage, as measured by DXA, compared to BMI (8). Similarly, in a cross-sectional analysis involving 81 young men, Corrêa et al. found that RFM exhibited stronger correlations with DXA and bioelectrical impedance analysis (BIA) results than BMI (27). Given these findings, we employed NHANES data from 2011 to 2018 to investigate the association between RFM and lumbar spine BMD.

In recent years, the relationship between obesity and osteoporosis (OP) has garnered increasing research attention. Visceral adipose tissue secretes pro-inflammatory cytokines (TNF-α, IL-1β), activating the RANKL/NF-κB pathway and stimulating osteoclastogenesis (21, 30–35). Upregulated fat mass and obesity-associated protein (FTO) expression in obese populations promotes the adipogenic differentiation of bone marrow stromal cells (BMSCs) while suppressing osteoblast activity (36). Obesity increases susceptibility to reactive oxygen species (ROS) accumulation, which inhibits the Wnt/β-catenin pathway—a critical osteogenic signaling cascade—and induces mitochondrial dysfunction (37). An intriguing study in diet-induced obese mice demonstrated that the clustered regularly interspaced short palindromic repeats system (CRISPR) interference targeting fatty-acid-binding protein 4 (Fabp4) in white adipocytes reduced bone resorption markers (CTX-1) and improved bone microstructure (38). Research by Chen et al. revealed that obesity-altered gut microbiota activates the Toll-like receptor 4 (TLR4) pathway via lipopolysaccharide (LPS), triggering senescence in bone marrow-derived macrophages (BMMs) and ultimately contributing to bone loss (39).

We explored potential explanations for the observed inflection point. Firstly, the complex interplay between fat tissue and estrogen regulation significantly influences bone metabolism. Estrogen promotes bone formation while inhibiting bone resorption (40, 41), and adipose tissue is a key site for aromatase activity, which facilitates estrogen production. Below the inflection point, extremely low fat levels reduce estrogen synthesis and increase pro-inflammatory factors. Concurrently, the combination of extremely low fat and insufficient mechanical loading on muscle and adipose tissue impairs bone formation (42, 43). This trend was consistently evident in smoothed curve analyses for both the subgroup with BMI < 25 and male participants. Secondly, RFM measurements tend to be less precise in individuals with lower body fat levels, with accuracy improving as fat percentage measured by DXA increases (8, 27). The left side of the inflection point may reflect this lower measurement reliability. Thirdly, for the female cohort, this inflection point appears physiologically implausible given the mean RFM value of 34.1037 in our study participants. We propose the following interpretation: The low inflection point may reflect methodological limitations. Weighted piecewise regression assumes an abrupt transition, whereas biological relationships are typically gradual. The underrepresentation of women with RFM < 20.5847% in our sample (*n* = 553) likely amplified statistical noise in the extremely low RFM group. Therefore, this threshold holds little practical significance for females, and future studies should conduct sex-stratified analyses to further validate such thresholds. Furthermore,the broader confidence intervals on the left side indicate reduced statistical power, likely due to smaller sample sizes in this range.

The leveling off observed around RFM = 42 in the subgroup with BMI > 30 kg/m^2^ is consistent with Frost’s mechanostat theory, which specifically describes bone’s adaptation to mechanical loading (44). This theory posits that bone density and strength are regulated in response to the physical forces applied upon it. However, the concurrent rise in fat mass, independent of the mechanical loading effects described by Frost, may introduce metabolic factors that could influence bone health, although the precise mechanisms are distinct. Interestingly, a similar plateau was noted at RFM = 35 in male participants, reinforcing our proposed hypothesis. It is noteworthy that the observed association between higher RFM and lower rates of smoking, alcohol consumption, and diabetes in our study population may reflect potential bias introduced by the higher proportion of female participants. Females inherently exhibit elevated RFM values (female sex coded as 1), yet typically demonstrate lower prevalence of smoking, alcohol use, and diabetes compared to males. Subgroup analyses highlighted intricate relationships between obesity, as defined by BMI, and BMD, underscoring the necessity for further research into the underlying mechanisms.

The observed inverse association between RFM and BMD carries significant implications for aging populations and public health practice. As global demographics shift toward older age groups, the dual burden of rising obesity rates and age-related bone loss presents a critical challenge. Our findings suggest that elevated adiposity—quantified by RFM—may accelerate bone mineral decline. Among the older adult, sarcopenic obesity and osteoporosis often coexist. Unlike traditional BMI measures, RFM more accurately reflects adipose distribution patterns that impact bone health, particularly by distinguishing fat mass from lean tissue. RFM’s non-invasive nature and ease of clinical application provide benefits compared to DXA for regular osteoporosis risk evaluation. Including RFM in current screening frameworks may improve risk prediction precision and support tailored treatment strategies. For example, focused adipose reduction and metabolic regulation approaches may reduce osteoporosis risk in populations with elevated RFM, allowing for proactive prevention and targeted management. This methodology could enhance the efficiency of screening processes while lowering fracture rates.

In summary, our study elucidates an inverse association between RFM and lumbar spine bone mineral density (BMD). The research possesses several strengths: a large sample size, a nationally representative cross-sectional design, adjustment for numerous confounding covariates to enhance the reliability of findings, and to our knowledge, it represents the first NHANES-based investigation exploring the relationship between RFM and lumbar spine BMD. However, this study is subject to limitations. The cross-sectional design precludes the examination of causal linkages between RFM and lumbar spine BMD. Furthermore, the data are derived exclusively from the U.S. population, which may limit generalizability to other demographic contexts. Additionally, lumbar spine BMD is influenced by multifactorial determinants, including postmenopausal effects on bone density in women, lifestyle variables, and other unmeasured confounders, which our analysis was unable to fully account for. Finally, the NHANES database does not include clinical diagnoses of osteoporosis through T-scores. Consequently, we were unable to determine the proportion of participants whose BMD values fell within the osteoporotic range. This limitation precludes direct inferences about the clinical implications of RFM on osteoporosis risk and may affect the generalizability of our findings to populations with confirmed osteoporosis. Future longitudinal studies incorporating dual-energy X-ray absorptiometry (DXA)-based osteoporosis diagnoses are needed to validate these preliminary findings in clinically defined populations.

## 5 Conclusion

Our study reveals a significant negative correlation between RFM and lumbar BMD in U.S. adults. These findings position RFM as a potential indicator for osteoporosis prevention. Maintaining optimal RFM levels may confer skeletal benefits. However, more in-depth studies are needed to validate this perspective (such as external data validation, among others).

## Data Availability

Publicly available datasets were analyzed in this study. This data can be found at: https://wwwn.cdc.gov/nchs/nhanes/Default.aspx.
